# *SAMHD1* knockout hiPSC model enables high lentiviral transduction efficiency in myeloid cell types

**DOI:** 10.3389/fgene.2025.1574545

**Published:** 2025-04-07

**Authors:** Huinan Li, Maliha Afroze, Gunisha Arora, Scot Federman, Kaivalya Shevade, Yeqing Angela Yang, Phuong Nguyen, Rustam Esanov, Laralynne Przybyla, Adam Litterman, Shawn Shafer

**Affiliations:** ^1^ Laboratory for Genomics Research, San Francisco, CA, United States; ^2^ Department of Biochemistry and Biophysics, University of California, San Francisco, San Francisco, CA, United States; ^3^ Target Discovery GSK, San Francisco, CA, United States

**Keywords:** myeloid cell, microglia, macrophage, CRISPR, functional genomics, IPSC

## Abstract

Recent advances in functional genomics tools have ushered in a new era of genetic editing to identify molecular pathways relevant to developmental and disease biology. However, limited model systems are available that adequately mimic cell states and phenotypes associated with human disease pathways. Here, we quantitatively analyzed the founder population bottleneck effect and demonstrated how the population changes from human induced pluripotent stem cells (hiPSCs) to hematopoietic stem cells and to the final induced macrophage population. We then engineered a key gene encoding an enzyme in the myeloid cell antiviral pathway-*SAMHD1-*knockout (KO) hiPSCs and characterized the hiPSC line with RNA-Seq and induced macrophages from two distinct protocols with functional analysis. We then generated *SAMHD1* KO CRISPR-dCAS9 KRAB hiPSCs through lentiviral transduction aiming to increase the efficiency of lentiviral mediated gene transfer. We demonstrated increased lentiviral transduction efficiency in induced macrophages, as well as microglia induced with two distinct protocols. This model allows for efficient gene knockdown, as well as large-scale functional genomics screens in mature hiPSC-derived macrophages or microglia with applications in innate immunity and chronic inflammatory disease biology. These experiments highlight the broad applicability of this platform for disease-relevant target identification and may improve our ability to run large-scale screens in hiPSC-derived myeloid model systems.

## Introduction

Macrophages and their tissue-resident counterparts, such as microglia in the brain, perform essential roles during development, homeostasis, and immunological disorders and are key cellular players in diseases such as neurological disorders in the central nervous system (Li and Barres, 2018; Park et al., 2022). The states of macrophages and microglia are altered in response to specific signaling pathways during development, disease, and in situations of acute or chronic inflammation (Locati et al., 2020; Keren-Shaul et al., 2017). In order to model human macrophages *in vitro*, primary cell cultures and humanized rodent models have been widely utilized to gain new insights into the initiation and progression of disease states (Park et al., 2022; Mass et al., 2023). However, such models often pose challenges when it comes to the genetic editing process as well as scalability. hiPSC-derived macrophages and microglia have addressed several of these challenges, including high efficiency of induction, faithful recapitulation of the innate gene signature, and response to physiologically relevant insults, which provide new avenues to model human disorders. Recently, with advances in the CRISPR technology, the capability and scalability of editing approaches have been greatly extended, allowing for modification of the human genome, epigenome, and transcriptome, followed by cell type-specific phenotypic readouts leading to a more comprehensive understanding of specific gene functions (Przybyla and Gilbert, 2022). This allows for functional phenotypic analyses to be conducted, but other challenges remain. Macrophages and microglia are known to be resistant to many forms of DNA delivery, including electroporation, transfection, and viral transduction (Maes et al., 2019). For this reason, hiPSC-induced macrophages and microglia are difficult to engineer on a large scale, particularly for introducing libraries of hundreds or even thousands of perturbations at a time.

Most cytokine-induced differentiation protocols do not investigate how the population changes from the founder cell population to the progenitor or mature differentiated states, but in many cases, the differentiated population is selected or sorted out, resulting in a population bottlenecking over the course of the differentiation trajectory (Abud et al., 2017; Muffat et al., 2016; van Wilgenburg et al., 2013). Using a transcription factor overexpression approach to deterministically drive differentiation provides an alternative route for generating a uniform population for functional genomic analysis. However, hiPSC lines engineered to overexpress transcription factors are difficult and time-consuming to generate (Dräger et al., 2022; Chen et al., 2021). In order to use a conventional cytokine-induced macrophage differentiation protocol in the context of functional genomic perturbation, we sought to develop a lentiviral strategy that improves macrophage and microglia viral infection efficiency. Human sterile alpha motif and HD domain-containing protein 1 (*SAMHD1*) degrade deoxynucleotide triphosphate (dNTP) into the 2′-deoxynucleoside (dN) and triphosphate subunits, maintaining the balance of intercellular dNTP pools and maintaining nucleotide homeostasis. In different cell types, SAMHD1 acts to either promote (e.g., dividing T cells (de Silva et al., 2013; Franzolin et al., 2013)) or restrict (e.g., nondividing macrophages (Diamond et al., 2004)) viral replication by maintaining homeostasis of the dNTPs. Previously, it was discovered that SAMHD1 activity protects monocytes and macrophages from viral infection by degrading dNTPs (Berger et al., 2011; Goujon et al., 2008). Inhibition of *SAMHD1* expression by RNAi or overexpression of a known repressor of SAMHD1 function viral protein X (VPX) has been shown to increase the efficiency of viral transduction in monocytes and macrophages (Laguette et al., 2011). THP-1 human monocytes (or THP1 differentiated macrophages) are widely used to study DNA sensing activity. This cell type expresses double-strand DNA sensors, including cyclic GMP-AMP synthase (cGAS), gamma-interferon-inducible protein (IFI-16), gamma-interferon-inducible protein 41 (DDX41), and leucine-rich repeat flightless-interacting protein 1 (LRRFIP1). A widely used THP-1 *SAMHD1* KO monocyte cell line (Oo et al., 2022) provides a useful model to investigate interferon regulatory factors (IRFs) and nuclear factor kappa-light-chain-enhancer of activated B cells (NF-κB) pathways, discover interaction between *SAMHD1* and other potential signaling proteins, and identify the role of SAMHD1 in innate immunity, tumorigenesis, or viral replication (Bonifati et al., 2016). However, to our knowledge, there is no published approach for generating *SAMHD1* KO hiPSC-induced macrophages.

We conducted a quantitative analysis of cell population diversity during induction to macrophages using two distinct protocols. This was done to examine the extent of population bottlenecks throughout hiPSC-derived macrophage (iMacrophages) differentiation. Through barcode labeling of the founder hiPSC population, we tracked the founder cell diversity to iMacrophages. We identified significant bottleneck effects in the hiPSC diversity during the iMacrophage induction process. This bottleneck effect encouraged us to explore alternative options that would allow for functional genomic studies in iMacrophages through lentiviral transduction. We then engineered a hiPSC line with *SAMHD1* gene knockout. The loss of *SAMHD1* has little effect on hiPSC health, and these cells can be induced into macrophages and microglia with multiple induction protocols. RNA-seq and functional tests on the differentiated populations indicate that knocking out *SAMHD1* also has little effect on the overall health of the induced cells, including iMacrophages and iMicroglia. In addition, we engineered an *SAMHD1* KO dCAS9 KRAB CRISPRi hiPSC line. We demonstrate that iMacrophages and iMicroglia have a dramatic increase in transduction efficiency with various lentiviral cargo types. We believe this model can be used for CRISPR functional genomic analysis, including introducing point mutation, knock out, and knock in of specific genes for functional genetic studies, even for large-scale CRISPR screens.

## Results

### Quantification of population diversity during macrophage differentiation

**FIGURE 1:**
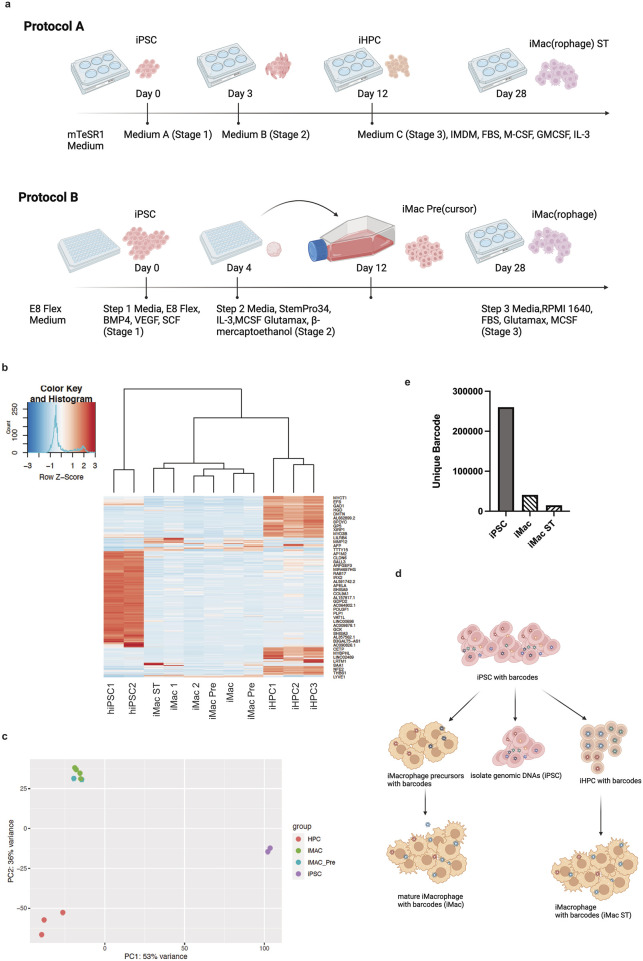
Founder cell population analysis of iMacrophages across two distinct protocols. **(a)** Schematic illustration of both iMacrophage induction protocols. Upper, hiPSCs cultured in mTeSR1 media were induced into iHPCs first with addition of media A and media B before being induced into iMacrophages (iMac ST) with media IMDM with FBS, supplemented with M-CSF, GMCSF, and IL-3; lower, E8 Flex-maintained hiPSCs were cultured in E8 Flex media supplemented with BMP4, VEGF, and SCF in a 384-well round bottom plate for 4 days before being induced into immature macrophages with StemPro34, supplemented with IL-3, M-CSF, GlutaMAX, and β-mercaptoethanol; the iMac Pre(cursors) were then transferred to a 6-well plate into iMacrophages (iMacs) with RPMI, FBS, GlutaMAX, M-CSF, **(b)** RNA sequencing data cluster analysis indicates duplicated samples in each stage expressing specific marker genes, **(c)** principle component analysis indicates hiPSCs cluster together, away from iHPCs, and iMacs of both inductions cluster together, as well as iMac precursors, **(d)** schematic illustration of sampling hiPSCs with barcodes across both inductions, and **(e)** hiPSC, iMac, and iMac ST populations of the individual barcode counts.

To understand the ability of differentiated macrophages to be used for functional genomics screening, we employed two different differentiation protocols. The first was a published protocol for obtaining iMacrophages (van Wilgenburg et al., 2013) (hereafter iMacrophages), and the second used a commercially available kit (Muffat et al., 2016; McQuade et al., 2018) (StemCell Technologies, hereafter iMacrophages ST). We compared two distinct differentiation protocols for macrophage differentiation from hiPSCs (Figure 1a) and analyzed their effects on gene expression and population bottlenecking upon transduction with a pooled viral library. To confirm that the two induction protocols generated the desired cell types, we performed bulk RNA sequencing and differential expression analysis (Figure 1b). Based on gene signatures, we were able to distinguish hiPSC, hiPSC-derived hematopoietic progenitor cells (iHPCs), and iMacrophages. iMacrophages from these two protocols were very similar, as confirmed using principal component analysis (Figure 1c). In the second protocol (Muffat et al., 2016; McQuade et al., 2018), iMacrophage precursors (iMacrophage Pre) were transcriptionally like fully differentiated iMacrophages (Figure 1b), consistent with the original description of this protocol (van Wilgenburg et al., 2013). To quantitatively understand the founder cell population identity changes throughout the whole induction, we performed these iMacrophage protocols using barcoded hiPSC populations. hiPSCs were infected with a virus containing unique barcodes, and then these cells were selected using puromycin and continued to proliferate until they were approximately 80% RFP- (red fluorescent protein) positive. These populations of cells were divided into three groups, each with an equal amount of hiPSCs as a founder cell population to ensure adequate representation of barcodes throughout the inductions (Figure 1d). One-third of hiPSCs were saved for sequencing as the initial founder cell population identity (hiPSC); the second third of hiPSCs were induced with a previously described protocol (van Wilgenburg et al., 2013). The final third of hiPSCs were induced into macrophages with another protocol previously described (Muffat et al., 2016; McQuade et al., 2018). According to the final data analysis, we were able to recover 260,050 unique barcodes in the founder population hiPSCs; after they were induced into iMacrophages with these two protocols, the barcode diversity decreased to 40,810 for the iMac population and to 14,998 for the iMac ST population. We also confirmed an overrepresentation of specific barcodes with a Shannon Evenness Index: hiPSC population, 0.909; iMacrophages, 0.763; and iMacrophages, ST 0.570, indicating the founder population drift phenomenon. This indicates that strong bottlenecks limit the hiPSC diversity upon differentiation with either protocol.

### Generation and characterization of SAMHD1 KO hiPSCs and differentiated cells

**FIGURE 2:**
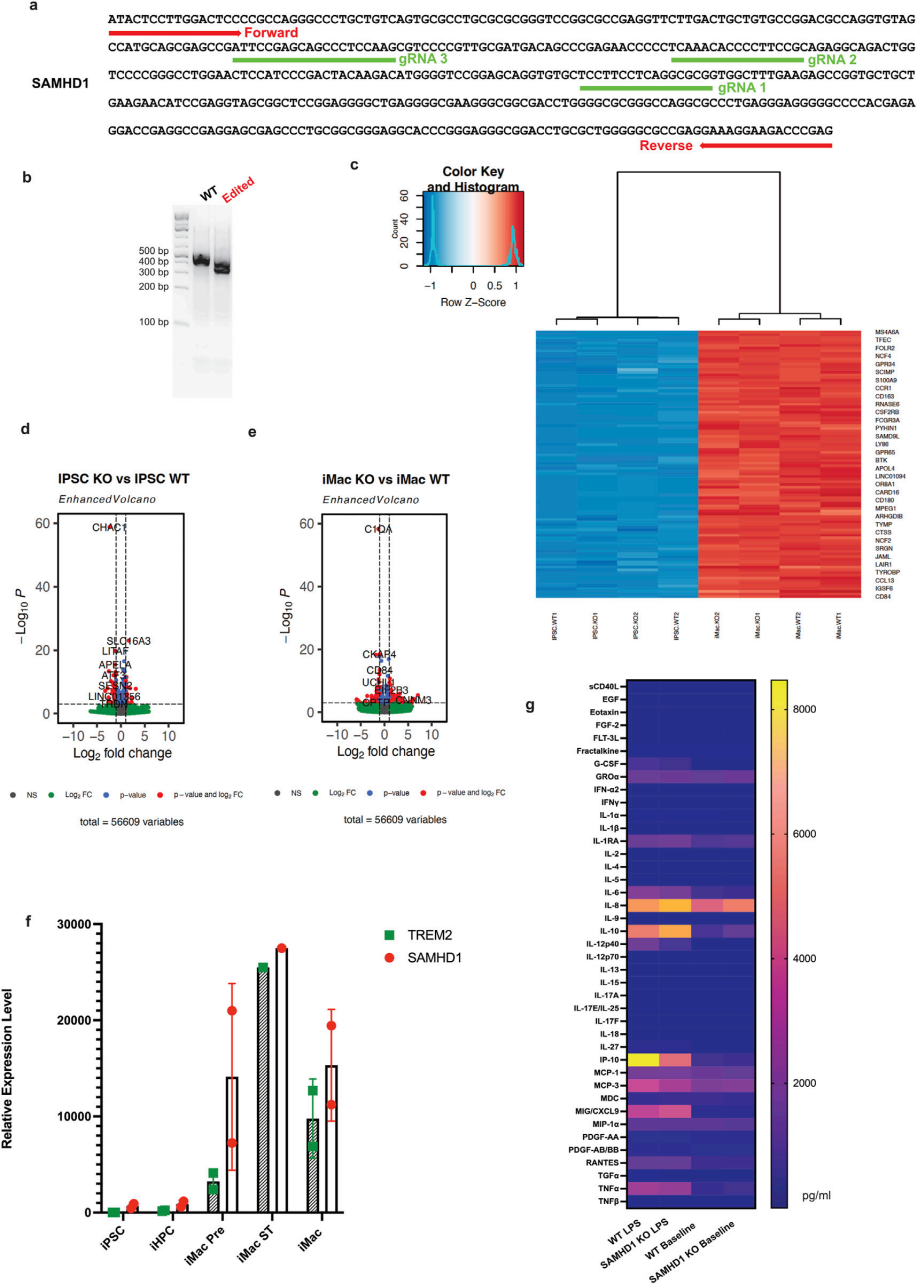
SAMHD1 knockout hiPSC line generation and characterization. **(a)** *SAMHD1* gene map sequence with forward and reverse primer sequences, as well as the positions of three guide RNAs, **(b)** PCR gel images indicating the truncation of the gene, **(c)** duplicated hiPSCs from two backgrounds and the induced macrophages show little difference, **(d)** volcano plot shows the differentially expressed genes between WT and *SAMHD1* KO hiPSCs, **(e)** volcano plot shows the differentially expressed genes between WT and *SAMHD1* KO iMacrophages, **(f)** comparison of *SAMHD1* gene relative expression from RNA sequencing in different cell types, with myeloid lineage marker TREM2, and **(g)** cytokine profiling using WT and *SAMHD1* KO iMacrophage prior to LPS treatment and after LPS treatment.

*SAMHD1* is a non-essential gene and has been demonstrated to inhibit viral infection in macrophages, so we hypothesized that *SAMHD1* mutant macrophages would be more permissive of lentiviral gene transfer (Coggins et al., 2020). To generate an *SAMHD1* knockout hiPSC cell line, we performed a Cas9 RNP electroporation with three synthetic single-guide RNAs targeting different regions of the *SAMHD1* exon1 into S2:1 iPS cells (Figure 2a). Gel electrophoresis analysis of a PCR amplicon of this exon confirmed the generation of a deletion that reduced the amplicon size by ∼100 base pairs (Figure 2b). Next, we used bulk RNA sequencing to examine the effect of *SAMHD1* knockout on gene expression in hiPSCs and differentiated iMacrophages (Figure 2c). In both hiPSCs and iMacrophages, the gene expression profiles were largely similar between WT and *SAMHD1* KO cells (Figures 2d, e). We also compared *SAMHD1* expression in different cell types from the RNA sequencing data, using microglia signature gene-triggering receptor expressed on myeloid cells 2 (*TREM2)* expression as a marker of macrophage differentiation. *SAMHD1* was expressed less in the hiPSC and iHPC stages, with increased expression in macrophage precursors and the highest level of expression in mature macrophages (Figure 2f). Furthermore, we profiled lipopolysaccharide (LPS), a known activator of macrophages (Meng and Lowell, 1997). Cytokine profiling of LPS-treated *SAMHD1* knockout and wildtype (WT) iMacrophages revealed that among the 48 cytokines we analyzed, only the C-X-C motif chemokine ligand 10 (CXCL10), also known as interferon gamma-induced protein 10 (IP-10), had a significantly different response, being lower in the *SAMHD1* knockout macrophages (Figure 2g). We then introduced a dCAS9-KRAB-BFP transgene (#188320, Addgene) (Heo et al., 2024) by lentiviral transduction and sorted blue fluorescent protein (BFP)-positive cells to isolate a pool of cells expressing the CRISPRi machinery.

### SAMHD1 KO CRISPRi (dCAS9-KRAB) hiPSC-induced microglia and macrophages have higher lentiviral transduction rates

**FIGURE 3:**
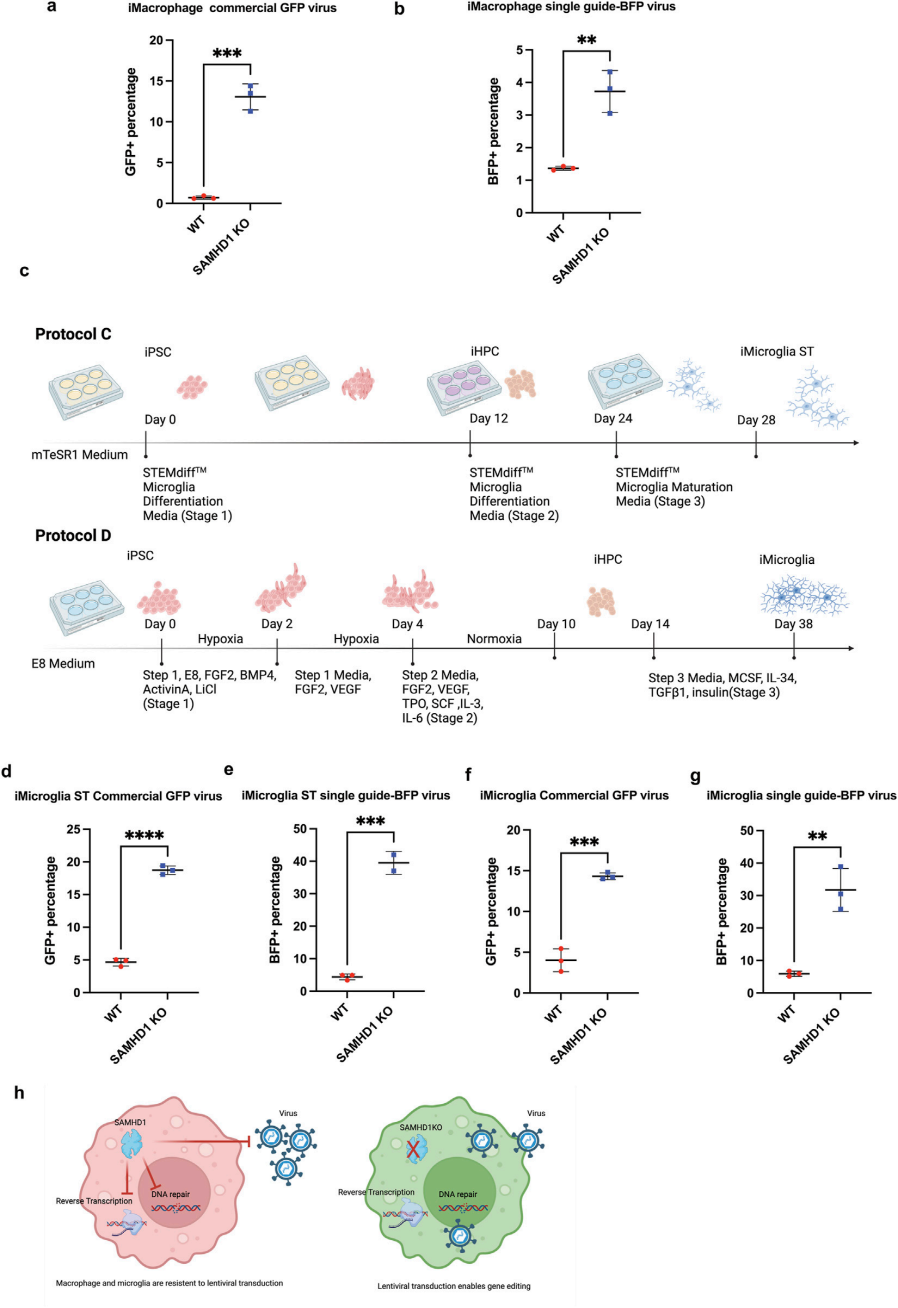
Comparisons between *SAMHD1* knockout CRIPSR dCAS9-KRAB hiPSC line-induced macrophages and microglia demonstrate increased lentiviral transduction efficiency. **(a)** GFP-positive cell percentage of iMac of WT and *SAMHD1* KO GFP lentivirus transduction, **(b)** BFP-positive cell percentages of iMacs of WT and *SAMHD1* KO BFP and CRISPRi single-guide lentivirus transduction, **(c)** schematic drawing of two protocols of iMicroglia induction, **(d)** GFP-positive cell percentages of iMicroglia of WT and *SAMHD1* KO GFP lentivirus transduction, **(e)** BFP-positive cell percentages of iMicroglia of WT and *SAMHD1* KO BFP and CRISPRi single-guide lentivirus transduction, **(f)** GFP-positive cell percentages of iMGL (hypoxia-induced microglia) of WT and *SAMHD1* KO GFP lentivirus transduction, **(g)** BFP-positive cell percentages of iMGL (hypoxia-induced microglia) of WT and *SAMHD1* KO BFP and CRISPRi single-guide lentivirus transduction, and **(h)** schematic summary of SAMHD1 KO myeloid cells increases lentiviral transduction efficiency and enables genetic modification.

In order to test the effect of *SAMHD1* on different cell types, we induced hiPSCs with *SAMHD1* KO as well as CRISPRi machinery into myeloid lineage cell types. First, we induced the hiPSCs into macrophages. hiPSCs were first induced into mesodermal lineage and into iHPCs (hematopoietic progenitor cells) in a serum-free and feeder-free condition. These iHPCs express CD34, CD45, and CD43. Then, iHPCs were matured into iMacrophages with the addition of cytokines, including IL-3, macrophage colony-stimulating factor (M-CSF), and granulocyte-macrophage colony-stimulating factor (GM-CSF). Microglia and macrophages are known to be challenging for lentiviral transduction. It is often suggested that high-titer viruses be used instead of mid- to low-titer viruses. To test the ability of these iMacrophages to be transduced upon maturation in both low-titer and high-titer viruses, we first produced a lentivirus containing a single-guide RNA as well as blue fluorescent protein (BFP) (Heo et al., 2024) at 5 × 10^6^ viral particle/mL low titer range and transduced *SAMHD1* knockout and WT iMacrophages with the optimized lentivirus concentration without triggering further immune response or cell death. One week after transduction, we observed 1.37% BFP+ cells in WT iMacrophages versus 3.73% in *SAMHD1* KO iMacrophages (Figures 3a, b). We observed a more marked difference in transduction efficiency using a commercially available high-titer green fluorescent protein (GFP) lentivirus with 0.73% WT iMacrophages exhibiting GFP positivity, while 13.07% of *SAMHD1* KO iMacs were GFP-positive (Figure 3b).

We then examined whether the above findings of increased transduction rate in *SAMHD1* KO iMacs applied to other phagocytic cell types derived from hiPSCs. We examined lentiviral transduction rates in three other cell types: iMacrophages ST, iMicroglia derived from a published differentiation protocol (Abud et al., 2017) (Figure 3c) (iMicroglia), and iMicroglia derived from a commercially available differentiation kit (McQuade et al., 2018) (Figure 3c) (Stemcell Technologies, hereafter iMicroglia ST). Cells derived from iMicroglia induction became over 80% CD11b- and CD45-double positive and over 50% TREM2-positive, reflecting a yolk sac-derived microglial cell state (Kierdorf et al., 2013). For both iMicroglia and iMicroglia ST, we observed strikingly higher transduction rates for *SAMHD1* KO cells with both the guide BFP and GFP lentiviruses (Figures 3d, e). For example, the increase in transduction in *SAMHD1* KO cells was nearly 10-fold using the GFP virus in iMicroglia ST, with the WT cells having a transduction rate of 4.41% versus 39.5% for *SAMHD1* KO cells Figures 3f, g). These data indicated that in three different phagocytic cell types derived from a *SAMHD1* KO hiPSC cell line using different differentiation protocols, *SAMHD1* KO allows cells to be transduced with markedly higher efficiency.

## Discussion

Macrophages and microglia play essential roles in development, tissue homeostasis, and immunological and neurological disorders (Li and Barres, 2018; Locati et al., 2020; Butovsky and Weiner, 2018). CRISPR genome editing and screens in rodent macrophages have provided information on genes that are essential for survival and response toward inflammation (Shi et al., 2022; Covarrubias et al., 2020). In addition, human primary cell lines, human immortal cell lines, and genetically engineered rodent models have been widely used in the research community and led to informative discoveries (Kamber et al., 2021). There are also many lines of evidence indicating human and rodent macrophages are vastly different in terms of disease phenotypes (Hodge et al., 2019). Therefore, *in vitro* models are needed as powerful tools for functional genomics purposes. Human hiPSCs can be differentiated into disease-relevant cell types on a large scale (Kampmann, 2020). Many cytokine-driven induction protocols recapitulate key features of innate cell populations and allow for human disease-relevant biology studies (Hasselmann and Blurton-Jones, 2020). Alternatively, through overexpressing transcription factors, hiPSCs can be directly induced into microglia-like cells (Dräger et al., 2022; Chen et al., 2021). These methods allow for a rapid induction of a homogenous population of microglia cells for large-scale functional genomic investigation (Dräger et al., 2022; Chen et al., 2021). However, it is known that such transcription factor overexpression strategies require extensive stem cell engineering and characterization by insertion of more than two gene coding regions. These strategies may, therefore, be challenging to use with specific hiPSC parental lines.

Macrophages and microglia are known to be refractory to commonly employed methods for gene transfer (Locati et al., 2020). This characteristic hinders the implementation of approaches that require gene manipulation and scalable transduction, including cell type-specific response and CRISPR editing, as well as large-scale CRISPR screens. *SAMHD1* is a key component during viral defense and is a non-essential gene (Laguette et al., 2011; Bonifati et al., 2016; Coggins et al., 2020). It has been demonstrated inhibiting the function of *SAMHD1* can increase viral transduction (Franzolin et al., 2013; Laguette et al., 2011; Mauney and Hollis, 2018).

In this study, we quantitively analyzed the initial founder cell population diversity across the induction with two distinct protocols (Muffat et al., 2016; van Wilgenburg et al., 2013; McQuade et al., 2018) for induction from hiPSCs into macrophages by assigning barcodes to individual hiPSCs. With the protocol that generated HPCs first and then matured them into macrophages (Muffat et al., 2016; McQuade et al., 2018), we found that the diversity of the barcodes remained similar until the HPC stage and plummeted to approximately one-tenth of the initial hiPSC population diversity by the mature macrophage stage. With the protocol that generated immature macrophages first (van Wilgenburg et al., 2013) and then matured them into macrophages, we found similar effects, with immature macrophage diversity similar to the original hiPSC diversity but dropped to about one-fifth of the initial diversity by the mature macrophage stage. This analysis is the first known quantitative analysis tracking barcode diversity during hiPSC induction into macrophages and provides quantitative evidence of a loss of library diversity during differentiation. Therefore, functional genomics screens performed by transducing libraries of genetic perturbations into hiPSCs will have only one-tenth of the diversity of the original transduction upon differentiation into macrophages. Our study highlights the need for approaches that bypass this bottleneck effect, which would require much higher screening coverage and could lead to dropouts and false-negative results in a screening experiment. Compared with previous literature that used exogenous Vpx addition to degrade *SAMHD1*, our method has less potential for cytotoxicity and phenotypic artifacts caused by the pleiotropic biological effects of Vpx addition (Gao et al., 2023; Hrecka et al., 2011). We have demonstrated that *SAMHD1* KO iMacrophage generation and iMicroglia generation, each using two protocols, exhibit increased viral transduction. These engineered cell lines, therefore, enable the transfer of guide RNAs with high efficiency at the macrophage and microglia stage, obviating the need for transduction at the hiPSC stage and bypassing this bottleneck effect. This finding was concordant across the four differentiation protocols, indicating that *SAMHD1* KO is a robust method for enabling genetic manipulation of multiple types of hiPSC-derived phagocytic cells that are refractory to lentiviral transduction. While *SAMHD1* KO had a large effect on the permissivity of phagocytic cells to lentiviral transduction, its overall biological effects were otherwise minimal (Figure 3h). By analyzing RNA sequencing and functional cytokine elaboration data from WT and *SAMHD1* KO macrophages, we observed minimal changes to gene expression and functionality, including expression of key markers of macrophage differentiation and elaboration of critical macrophage-derived cytokines in response to LPS stimulation.

Using our *SAMHD1* KO approach provides a method for genetic manipulation of several human phagocytic cell types, overcoming the difficulty in achieving diverse pools of genetically perturbed cells in these systems. In the future, we anticipate that this approach will empower large-scale genetic screens in relevant cellular models of human immunological and neurodegenerative diseases. *SAMHD1* acts as a defense mechanism in myeloid cells. We observe that after knocking out *SAMHD1*, iMacrophages exhibit impaired cytokine response, including IP-10. This cell model is less optimal for studies focusing on understanding signaling pathways in viral defense with cytokine-related investigation.

## Materials and methods

### Image generation

Images are generated using bioRender (Created at https://BioRender.com).

### *SAMHD1* KO and CRISPRi line engineering

The human biological samples were ethically sourced, and their research use was in accordance with the terms of the informed consent under an IRB/EC-approved protocol. The hiPSC line (S2:1) background used in this study was generated from a healthy donor peripheral blood mononuclear cells (PBMCs) via Sendai virus reprogramming by Takara Bio Inc. at GlaxoSmithKline request. To generate the SAMHD1 KO hiPSC line, a gRNA pool from Synthego targeting the first exon of the SAMHD1 gene (gRNA1: AAA​GCC​ACC​GCG​CCU​GAG​GA, gRNA2: UCU​GCG​GAA​GGG​GUG​UUU​GA, gRNA3: CUU​GGA​GGG​CUG​CUC​GGA​AU) was used. Cells were dissociated with Accutase, and 500 K cells were resuspended in 20 μL of P3 solution (Lonza) and 5 μL of RNP mix (40 pmol of Cas9 and 300 pmol of gRNA mix) for a nucleofection reaction (Lonza Nucleofector, code CA137). Post nucleofection, cells were seeded onto Geltrex (Thermofisher, A1413201)-coated plates and incubated in mTeSR1 media in the presence of the ROCK inhibitor (10 μM) overnight. Three days later, cells were used for DNA extraction (Lucigen QuickExtract) and PCR to confirm knockout. For PCR, primers spanning gRNA binding sites were designed (Forward: ATA​CTC​CTT​GGA​CTC​CCC​GC, Reverse: CTC​GGG​TCT​TCC​TTT​CCT​CG), and the reaction was performed using Ultra II Q5 DNA polymerase (NEB). PCR products were run on 1% agarose gel for visualization. To introduce CRIPSRi machinery for screening, the SAMHD1 KO hiPSC line was transduced with a lentivirus encoding for dCas9-KRAB-P2A-mCherry at a multiplicity of infection (MOI) of 0.1 overnight in the presence of polybrene (1 μg/mL). After transduction, hiPSCs were expanded, and mCherry-positive cells were sorted by FACS.

### Barcoding hiPSC populations

Barcoding hiPSC populations were made according to the manufacturer’s instructions (CloneTracker XP™ Lentiviral Barcode Libraries). Pilot experiments were conducted to determine cell doubling time and to generate a killing curve. After viruses were made with HEK 293 cells, hiPSCs were transduced with the library. The second day verified the 12% transduction rate. hiPSCs were selected with puromycin until more than 90% were selected. hiPSCs were passaged until reaching around 4.5 million in total to proceed to induction. These populations of cells were divided into three groups, each with 1.5 million hiPSCs, to ensure adequate representation of barcodes throughout the inductions (Figure 1d). Of a total of 4.5 million cells, the first 1.5 million cells were saved for sequencing as the initial founder cell population identity (hiPSCs). The second 1.5 million cells were induced with a previously described protocol: briefly, hiPSCs on Day 20, 1.5 million macrophage precursors in a T175 flask were harvested again for sampling analysis, and 1.2 million macrophages cultured in a 6-well plate with Step 3 media were harvested for sampling analysis; on Day 25, a total of 4 million mature macrophages (iMacrophage) were divided into 2 million and 2 million cells for sample analysis. The third 1.5 million cells were induced into macrophages with another protocol previously described. Briefly, on Day 0, 1.5 million hiPSCs were cultured with media A in 6-well plates. On Day 2, half of media A was changed; on Day 3, media B was switched, and half of the media was changed every other day. On Day 12, HPC started to float and could be harvested and transferred to another 6-well plate for macrophage culture. On Day 16, the total HPC harvest was 3 million: 1.5 million were saved for sample analysis, and the other 1.5 million HPCs were continued to be cultured into macrophages. On Day 23, 1.4 million immature macrophages were harvested for sampling analysis, and on Day 37, another 1.4 million immature macrophages were harvested for sampling analysis. Finally, on Day 44, the remaining 1 million macrophages were harvested for sampling analysis (iMacrophage ST). Genomic DNAs were isolated with a 740954.20 NGS Prep Kit for Barcode Libraries in pScribe (CloneTracker XP™, LNGS-300), which was used for genomic library amplification and all the primers and reagents for the first and second amplifications and sequencing of the genomically integrated barcodes.

### Barcode counting

For each barcoded sample, FASTQ files were inspected to determine the BC14/BC30 sequence present at the defined location within the sequenced read, using perfect matching. The number of unique barcodes matching the expected barcodes from CloneTracker were counted, as well as numbers derived from earlier cellular stages. The Shannon Index/Maximum/Evenness was calculated for each sample.

### Code availability

The code used in this study is available from the authors upon written request.

### Statistical analysis

The statistical significance of differences between groups was determined with unpaired T-tests. Tests were deemed statistically significant at the *p < 0.05, **p < 0.01, ***p < 0.001, and ****p < 0.0001 levels.

### iMacrophage differentiation

Macrophages were induced with two independent previously published protocols (Muffat et al., 2016; van Wilgenburg et al., 2013). For the first method listed as Protocol A, the HPC induction followed the manufacturer’s protocol. Briefly, on Day −1, hiPSCs were plated at 10–20 times lower plating density onto a Matrigel-coated 6-well plate with 2 mL mTeSR media. On Day 0, the mTeSR media was removed, and 2 mL of Media A was added. On Day 2, 1 mL media was removed and replaced with 1 mL fresh Media A. On Day 3, all media was removed, and 2 mL of Media B was added. On Day 5, 1 mL of Media B was removed, and 1 mL of fresh Media B was added. A 1 mL media change was performed on Days 5, 7, and 10. On Day 12, all cells were transferred to a PDL-coated plate, and cells were fed with Media C [500 mL IMDM (Gibco), 10% defined FBS (Gibco), 5 mL penicillin/streptomycin (Gibco)), 20 ng/mL of hIL3 (Peprotech), 20 ng/mL of hGMCSF (Peprotech), and 20 ng/mL of hM-CSF (Peprotech)]. It took an average of 3–4 weeks for the macrophages to mature. For the second method, briefly, from Day 0–4, 10,000 cells (50 μL) were cultured in Step 1 media [Essential 8 Flex, 10uM ROCK Inhibitor, 50 ng/mL BMP-4, 20 ng/mL SCF, 50 ng/mL VEGF]. On Day 4, embryo bodies were formed. These embryo bodies were transferred to a 0.1% gelatin-coated T175 flask in media Step 2 [X-vivo (10639011, LONZA) or StemPro 34 (10639011, Gibco), 25 ng/mL IL-3, 100 ng/mL M-CSF, 2 mM GlutaMAX, and 55um ß-mercaptoethanol], and media was changed every 7 days until Day 18. On Day 18, floating precursors were transferred to a 6-well plate, each well with approximately 1.2 million cells in Step 3 media (RPMI1640 + 10% FBS, 2 mM GlutaMAX and 100 ng/mL M-CSF). On Day 25, mature macrophages were used for further analysis.

### iMicroglia ST and iMicroglia differentiation

iMicroglia ST (Protocol C) were induced following the manufacturer’s instructions with a combination of induction kit and maturation kit (#100-0019 and #100-0020, Stem Cell Technologies) based on a previous publication (McQuade et al., 2018). Briefly, on Day 0, to hiPSCs growing in a 6-well plate was added STEMdiff™ Microglia Differentiation (#100-0019) with half media addition every other day till Day 12. On Day 12, the cell suspension was collected and centrifuged at 300 g for 5 min. Cells were then resuspended in STEMdiff™ Microglia Differentiation Media and were seeded into Matrigel (Corning) coated plates until Day 24 with media addition every other day. On Day 24, cells were collected and centrifuged at 300 g for 5 min. Cells were then resuspended in STEMdiff™ Microglia Maturation Media (#100-0020, Stem Cell Technologies). On Days 28–34, cells started to mature into iMicroglia ST and became less proliferative.

iMicroglia were differentiated as previously described (Abud et al., 2017) (Protocol D). Briefly, hiPSCs were cultured in mTeSR1 (Stem Cell Technologies) media on Matrigel (Corning) coated 6-well plates (Corning). When hiPSCs reached approximately 80% confluency, they were dissociated using ReleSR (Stem Cell Technologies) and collected in 15 mL canonical tubes, followed by centrifugation for 5 min at 300×g and counted using trypan blue (Thermo Fisher Scientific). Typically, 200,000 cells/well were resuspended in mTeSR1 in low adherence 6-well plates (Corning). For Day 1 to Day 10, cells were cultured in DM [50% IMDM (Thermo Fisher Scientific), 50% F12 (Thermo Fisher Scientific), ITSG-X 2% v/v (Thermo Fisher Scientific), L-ascorbic acid 2-phosphate (64 μg/mL, Sigma), monothioglycerol (400 mM, Sigma), poly (vinyl) alcohol (PVA) (10 mg/mL, Sigma), GlutaMAX (1X, Thermo Fisher Scientific), chemically defined lipid concentrate (1X, Thermo Fisher Scientific), and non-essential amino acids (Thermo Fisher Scientific)]. At Day 0, embryoid bodies (EB) were gently collected, centrifuged at 100×g, and resuspended in DM medium supplemented with 1 μM ROCK inhibitor, FGF2 (50 ng/mL, Thermo Fisher Scientific), BMP4 (50 ng/mL, Thermo Fisher Scientific), Activin-A (12.5 ng/mL, Thermo Fisher Scientific), and LiCl (2 mM, Sigma), then incubated in a hypoxic incubator for 48 h (5% O_2_, 5% CO_2_, 37°C). On Day 2, cells were gently collected, and the media was changed to DM medium supplemented with FGF2 (50 ng/mL, Thermo Fisher Scientific) and VEGF (50 ng/mL, PeproTech) and returned to the hypoxic incubator for another 48 h. On Day 4, cells were gently collected, and media changed to DM supplemented with FGF2 (50 ng/mL, Thermo Fisher Scientific), VEGF (50 ng/mL, PeproTech), TPO (50 ng/mL, PeproTech), SCF (10 ng/mL, Thermo Fisher Scientific), IL6 (50 ng/mL, PeproTech), and IL3 (10 ng/mL, PeproTech) and incubated in a normoxic incubator (20% O_2_, 5% CO_2_, 37°C). On Day 6 and Day 8, 2 mL of Day 4 media was added to each well. On Day 10, cells were collected, counted using trypan blue, and frozen in Bambanker (Nippon Genetics) in aliquots of 500,000–1,000,000 cells. To start iMicroglia differentiation, cells were thawed, washed 1× with PBS, and plated at 100,000–200,000 cells per well in 6-well plate coated with Matrigel in iMicroglia media [(DMEM/F12 (Thermo Fisher Scientific), ITS-G (2% v/v, Thermo Fisher Scientific), B27 (2% v/v, Thermo Fisher Scientific), N2 (0.5% v/v, Thermo Fisher Scientific), monothioglycerol (200 mM, Sigma), GlutaMAX (1X, Thermo Fisher Scientific), non-essential amino acids (1X, Thermo Fisher Scientific)] supplemented with M-CSF (25 ng/mL, PeproTech), IL-34 (10 ng/mL, PeproTech), and TGFB-1 (50 ng/mL, PeproTech). Cells were fed every 2 days and replated on Day 22. On Day 30, cells were collected and replated in iMGL media supplemented with M-CSF (25 ng/mL, PeproTech), IL-34 (10 ng/mL, PeproTech), TGFB-1 (50 ng/mL, PeproTech), CD200 (100 ng/mL, VWR), and CX3CL1 (100 ng/mL, PeproTech). Cells were used on Day 40 for functional and transcriptomic assays.

### Bulk RNA-seq analysis

Total RNA was extracted from cell pellets for duplicate samples using the Monarch Total RNA Miniprep Kit (New England BioLabs). RNA quality and concentration were checked on Tapestation (Agilent) with RNA ScreenTapes (Agilent). Bulk RNA-seq libraries were prepared using the QuantSeq 3′ mRNA-seq Library Prep Kit FWD (Lexogen). Final libraries were quantified with Tapestation (Agilent) with D1000 HS ScreenTapes (Agilent) and Qubit dsDNA HS reagents (Thermo Fisher Scientific). Sequencing was performed on NextSeq 550 (Illumina) for single-end 100-bp reads. Reads were first trimmed to remove the sequencing adapters using bbduk (https://sourceforge.net/projects/bbmap/) and then aligned to the hg38 reference human genome using STAR (Dobin et al., 2013). Reads mapped to the genome were then assigned to genomic features (genes) using feature counts and the Rsubread package (Liao et al., 2019). Differential expression analysis was performed by using the negative binomial distribution from the R package DESeq2 (Love et al., 2014). Principle components analysis (PCA) was performed to visualize the variance in the samples. PCA plots and volcano plots showing differentially expressed genes were plotted using the ggplot2 package (https://tidyverse.github.io/ggplot2-docs/authors.html). P-value and log fold change cutoffs used to filter the differentially expressed gene lists were 0.0001 and ± 1, respectively.

### Cytokine profiling

Approximately 300,000 cells/well of a 12-well plate with macrophages of wildtype (WT) and *SAMHD1* KO were cultured with Media C [500 mL IMDM (Gibco), 10% defined FBS (Gibco), 5 mL penicillin/streptomycin (Gibco)), 20 ng/mL of hIL3 (Peprotech), 20 ng/mL of hGMCSF (Peprotech), and 20 ng/mL of hM-CSF (Peprotech)]. 100 ng/mL LPS was added to both WT and *SAMHD1* KO macrophages. Aliquots (50 μL) of culture media of WT, WT treated with LPS and *SAMHD1* KO, and *SAMHD1* KO treated with LPS were harvested, flash frozen, and assayed for 48-plex protein levels by quantitative immunoassay (Eve Technologies).

### Lentiviral production

Lenti-X cells were seeded at 18,000,000 cells per plate in 15 cm dishes in 20 mL media (DMEM, 10% FBS) and incubated overnight at 37°C, 5% CO_2_. The next morning, 8 mg sgRNA library plasmid, 4 mg psPAX2 (Addgene #12260), 4 mg pMD2.G (Addgene #12259), and 80 mL lipofectamine 2000 (Invitrogen) were mixed into 1 mL serum-free OptiMEM (GIBCO), vortexed, and incubated for 20 min at RT and added to the cells. At 72 h post-transfection, the supernatant was harvested, passed through 0.45-μm filters (Millipore, Stericup), and aliquots were stored at 80°C. Samples of 6–8 × 10^6^ functional viruses per ml have been routinely made in the lab. Commercial virus (LentiBrite GFP Control Lentiviral Biosensor, 17–10387, Millipore Sigma) was purchased and used at the manufacturer’s suggestion. One vial contained 25 µL of lentiviral particles at a minimum of 3 × 10^8^ infectious units (IFU) per ml.

## Data Availability

The data presented in the study are deposited in the Gene Expression Omnibus repository, accession number (GEO)-GSE289724 for the RNASeq and (GEO)-GSE289725 for the barcode_count.
